# Psychological resilience in patients with Friedreich ataxia: a 6-year longitudinal analysis

**DOI:** 10.1186/s42466-026-00499-z

**Published:** 2026-05-19

**Authors:** Ruth Eumann, Janna Krahe, Imis Dogan, Ana Sofia Costa, Jörg B. Schulz, Stella A. Lischewski, Kathrin Reetz, the FACROSS Study Group, Sandro Romanzetti, Ravi Dadsena, Maximillian Praster, Miguel Pishnamaz, Kerstin Konrad, Thomas Clavel, Vera Jankowski, Joachim Jankowski, Oliver Pabst, Nikolaus Marx, Julia Möllmann, Malte Jacobsen, Katharina Marx-Schütt, Juergen Dukart, Simon B Eickhoff, Ralf-Dieter Hilgers

**Affiliations:** 1 Department of Neurology, RWTH Aachen University Aachen, Pauwelsstrasse 30, 52074 Aachen, Germany; 2 JARA-BRAIN Institute Molecular Neuroscience and Neuroimaging, Forschungszentrum Jülich GmbH, Jülich, Germany

**Keywords:** Friedreich ataxia, Psychological resilience, COVID-19 pandemic, Longitudinal development

## Abstract

**Background:**

Friedreich ataxia is a debilitating multisystem neurodegenerative disorder. Depression is more prevalent in individuals with Friedreich ataxia (14%-36%) than in the general European population (8.5%). While psychological resilience is known to influence mental health in other chronic conditions, its role in Friedreich ataxia remains underexplored.

**Objectives:**

We aimed to compare psychological resilience between individuals with Friedreich ataxia and healthy controls, assess associations with clinical or demographic characteristics, depression, and anxiety, and explore changes in resilience longitudinally.

**Methods:**

Participants were drawn from the Aachen site of the European Friedreich Ataxia Consortium for Translational Studies (EFACTS) registry. Resilience was measured using the Wagnild and Young RS-25 questionnaire. Depression and anxiety were assessed using the Hospital Anxiety and Depression Scale and Beck’s Depression Inventory II. Student’s t-tests were used to compare groups. Pearson correlations assessed associations between resilience, depression, and anxiety scores. Univariate and multiple linear regressions were conducted to identify factors associated with resilience. Longitudinal resilience trends were evaluated graphically, and a within-person analysis was conducted using paired-T-tests in participants with available data. Missing data were imputed in 10% of cases.

**Results:**

Overall, 61 participants (43 with Friedreich ataxia, 18 controls) were followed for up to six years. Median age was 30 years for individuals with Friedreich ataxia and 28 years for controls; 58.1% and 61.1% were female, respectively. At baseline, resilience scores were significantly lower in individuals with Friedreich ataxia (135.1 ± 25.3) than in controls (151.5 ± 12.2; p < 0.05). Resilience was strongly negatively correlated with depression (r = -0.85, p < 0.001) and moderately with anxiety (r = -0.65, p < 0.001). We observed an apparent decline in resilience in patients during the COVID-19 pandemic albeit not statistically significant in the within-person subgroup analysis (pre-COVID-19 143.1 vs 131.2 post COVID-19; p = 0.07, n = 18), possibly due to low sample size.

**Conclusions:**

Individuals with Friedreich ataxia display reduced resilience compared to healthy controls, which is associated with greater levels of depression and anxiety. Resilience remained stable over the disease course but appeared to decline during the COVID-19 pandemic, underlining the importance of strengthening resilience and mental well-being in this population.

**Supplementary Information:**

The online version contains supplementary material available at 10.1186/s42466-026-00499-z.

## Introduction

Friedreich ataxia is a debilitating, multisystem neurodegenerative disease and the most common hereditary ataxia in individuals of predominantly Caucasian or Indian ancestry. Its global prevalence is estimated at 1 in 50,000 people although this number varies considerably between different countries [1–3]. It follows an autosomal recessive inheritance pattern and the first manifestations most frequently appear around puberty [4]. However, early-onset and late-onset variants occur. [4] Most patients with Friedreich ataxia have homozygous GAA trinucleotide repeat expansions in the frataxin (*FXN*) gene [5]. There is an inverse correlation between the GAA repeat expansion length and the age of symptom onset and prognosis [6]. Symptoms include ataxia, dysarthria, scoliosis, deformities of the foot, hypertrophic cardiomyopathy, dysphagia, diabetes mellitus, depression, and anxiety [5, 7]. Due to the progressive nature of the disease, patients with Friedreich ataxia gradually lose independence in their daily activities, and their life expectancy is significantly reduced [8].

The estimates for the prevalence of depression in patients with Friedreich ataxia range from 14 to 36%, which is higher than the 8.5% reported in the general population in Europe [7, 9, 10]. While the aetiology remains unclear, current evidence suggests that depressive symptoms in Friedreich ataxia likely reflect a multifactorial process, shaped by disease-related neuropathology, such as cerebellar involvement, as well as individual psychological traits and the cumulative burden of living with a progressive condition. [9, 11].

Resilience may play a large part in the psychological well-being of individuals with Friedreich ataxia, as they navigate the challenges posed by the progressive nature of the disease. The American Psychological Association defines psychological resilience as ‘the process and outcome of successfully adapting to difficult or challenging life experiences’ [12]. Most theories agree that psychological resilience is not a static trait, but rather a dynamic process that changes over time and can be influenced by various factors [13]. It is understood to be a protective measure against adverse reactions to stress [13]. Accordingly, negative associations between depression and resilience have been reported [14, 15]. Furthermore, resilience has been outlined as a mediating factor of depression and anxiety in other chronic conditions [16–19]. A systematic review on resilience in chronic diseases revealed that patients with low resilience scores have a diminished ability to cope with stress and other challenges that arise during disease progression [18]. To our knowledge, no studies have researched resilience in individuals with Friedreich ataxia.

Our aim was to explore psychological resilience in individuals with Friedreich ataxia and compare it with that of healthy individuals. We also sought to understand how various factors, including demographic characteristics, disease-related variables, and socioeconomic conditions influence resilience. Additionally, we explored the relationships among resilience levels, anxiety, and depression, as well as how resilience may evolve.

## Methods

### Participants and Procedures

Patients with Friedreich ataxia attending our outpatient clinic in Aachen, Germany and who participated in the EFACTS (European Friedreich Ataxia Consortium for Translational Studies) registry, a European multinational prospective cohort study, were included. The EFACTS registry is described in detail elsewhere [4, 20, 21]; briefly, patients with genetically confirmed Friedreich ataxia and healthy controls attended annual visits during which demographic and clinical data were collected. Since 2017, EFACTS participants enrolled in Aachen also completed measures related to psychological resilience. Participants were enrolled progressively over time. Owing to the COVID-19 pandemic, the assessment of healthy controls paused between Spring 2020 and Summer 2021, whilst the assessment of patients continued.

### Measures

We used the German adaptation of the Wagnild and Young Resilience Scale (RS-25) to assess resilience [22, 23]. Participants were asked to rate 25 statements on a seven-point Likert scale, ranging from 1—strongly disagree to 7—strongly agree. Scores range from 25 to 175, with high scores indicating high resilience. The RS-25 is structured to assess five elements that collectively make up the “Resilience Core”, which includes “equanimity”, “perseverance”, “self-reliance”, “meaningfulness” and “existential aloneness” [23]. While the original version included two subscales — personal competence and acceptance of self and life —, this two-factor model could not be replicated in the German adaptation. Therefore, only the total score was used in this study. The German RS-25 demonstrated strong reliability (internal consistency Cronbach’s α = .95) [22].

To evaluate depression and anxiety, the German version of the Hospital Anxiety and Depression Scale (HADS) was used [24, 25]. The scale generates two subscores to assess anxiety (HADS-A) and depression (HADS-D) separately. Subscale scores below 7 points indicate no clinically significant anxiety or depression symptoms. Subscores of 8—10 are classified as ‘borderline’ and may indicate mild symptoms, whereas subscores between 11 and 21 points suggest clinically significant symptoms of anxiety or depression.

We also used the German adaptation of the revised Beck’s Depression Inventory(BDI-II) [26]. This self-report scale, which has 21 items, aims to assess the severity of depressive symptoms. Each item is rated on a four-point scale ranging from 0—3. Scores from 0–12 indicate no depression, scores from 13–19 indicate mild depression, scores from 20–28 indicate moderate depression, and scores from 29–63 indicate severe depression.

### Data handling and analysis

We used multiple data imputation with five imputations to impute missing values, as it has been proven superior to other methods used to address missing data at item level [27]. We reported pooled results where possible; otherwise, “Rubin’s Rules” were used to calculate mean and between-imputation variance where necessary [28].

Welch’s unequal variances t test, an adaptation of the independent samples t test, was used to determine differences in resilience scores between patients with Friedreich ataxia and healthy controls. This approach was chosen due to a violation of the assumption of homogeneity of variances, as indicated by Levene’s test for Equality of Variances [29].

We used univariate linear regression to assess the relationships between resilience scores and demographic, disease-related, lifestyle-related and socioeconomic factors. We determined Pearson’s correlation coefficient to investigate the relationship between resilience scores and depression assessment scores. The assumption of linearity was assessed via scatterplots with a superimposed regression line. Due to an unbalanced sample size resulting from lower response rates on the HADS and BDI-II, we excluded missing values pairwise. Ultimately, 31/43 (72%) of people with Friedreich ataxia filled out the complete HADS and two more completed only the anxiety assessment of the HADS. The evolution of the mean resilience score of patients with Friedreich ataxia over time was assessed graphically. The univariate regression analyses as well as the correlation analyses with HADS and BDI-II were only conducted in the cohort of patients with Friedreich ataxia. Healthy controls were included for between group comparisons and descriptive graphical presentation. We conducted a paired-samples t-test to investigate changes in resilience scores before and after the COVID-19 pandemic within subjects for participants with data on at least two visits. We used the last resilience score obtained pre-2020, before COVID-19 lockdowns in Germany, and the most recent resilience score obtained in either 2022 or 2023. The assumption of normality was assessed using the Shapiro-Wilks-Test.

Outliers were identified via the interquartile range (IQR) method, where values falling below Q1—1.5 × IQR or above Q3 + 1.5 × IQR were considered outliers. We used Leverage and Cook’s distance values to determine the impact of the outlier on regression results.

The data were analysed via IBM SPSS Statistics for iOS Version 28.0 (released 2021, IBM Corp., Armonk, NY). The data are reported as mean, standard deviation, range or frequency and rounded as appropriate. P values < 0.05 were considered significant and two-tailed P values were reported.

## Results

### Baseline characteristics

Our study population comprised 61 participants, 45 of whom were patients with Friedreich ataxia and 18 of whom were healthy controls. Two individuals with Friedreich ataxia were excluded because they did not have any valid demographic data at baseline and analyses were ultimately performed on 43 individuals with Friedreich ataxia. Baseline was defined as the first time participants completed the RS-25 survey. At baseline, the median age of participants was similar between the two groups, with patients at 30 years and healthy controls at 28.5 years. Both groups had a comparable sex distribution (approximately 59% female overall). The baseline demographic data are reported in Table 1. Upon examining the data, we noticed missing data in the RS-25 score in 10.1% of the cases, for a total of 47 out of 4403 values. We generated a missing value pattern and found no monotonicity or concentration; thus, we decided to handle the data as missing at random [30].

**Table 1:** Baseline characteristics

Characteristics		Patients (n = 43)	Healthy controls (n = 18)	Overall (n = 61)
Age (years)		30 (23—38)	28.5 (25—40.25)	29 (24—38.5)
Sex ( female)		58.1%	61.1%	59.0%
Education (years)*		13 (10—15)	17.5 (15—18.5)	15 (11.25—17)
Age of Onset*		13.5 (10—18.25)	NA	NA
Children		20.9%	16.7%	19.7%
Employed		37.2%	72.3%	47.5%
Employment (part vs full time)		16.3% vs 20.9%	55.6% vs 16.7%	27.8% vs 19.7%
Scoliosis*		79.1%	NA	NA
Wheelchair Bound (yes vs no)		46.5%	NA	NA
GAA -Repeats Shorter Allele		567(445—712)	NA	NA
SARA Score**		19 (12.25—30)	NA	NA
ADL Score		14 (9—22)	NA	NA
HADS Total Score↑		10 (4—14)	3 (1.5—5.5)*	6 (3—11)
	HADS Anxiety Subscore	6 (2—8)	2 (1—3.5)	4 (2—6)
	HADS Depression Subscore	4 (2—7)	1 (0—2.5)	2.5 (1—5)
BDI-II Score Ø		6 (2.5—10.5)	0 (0—1)**	3 (0—8)
Disability stage		5 (2—6)*	NA	NA
	Mild	4.7%		
	Moderate, unable to run	20.9%		
	Severe, walking with 1 stick	7.0%		
	Walking with 2 sticks	20.9%		
	Unable to walk	46.5%		
Ethnic Group				
	Caucasian	90.7%	88.9%	90.2%
	African	2.3%	5.6%	1.6%
	Asian	7.0%	5.6%	6.6%
Education (ISCED levels)*		3 (1—6)*		
	0—nursery	0.0%	0.0%	0.0%
	1—primary school	9.3%	0.0%	6.7%
	2—lower secondary school	27.9%	0.0%	21.7%
	3—upper secondary school	46.5%	38.9%	43.3%
	4—college of further education	7.0%	5.6%	6.7%
	5—university	4.7%	55.6%	20.0%
	6—tertiary studies	2.3%	0.0%	1.7%
Marital Status				
	1- single	53.5%	33.3%	45.9%
	2—married	16.3%	5.6%	13.1%
	3—widowed	0.0%	0.0%	0.0%
	4—divorced	4.7%	5.6%	4.9%
	5—seperated	4.7%	0.0%	3.3%
	6 -in a relationship	20.9%	55.6%	32.8%

Data are median (IQR) or n/N% if not otherwise indicated NA: Not Applicable, SARA: Scale for the Assessment and Rating of Ataxia, ADL: Activities of Daily Living, HADS: Hospital Anxiety and Depression Scale, BDI-II: Beck’s Depression Index II. *Missing Data for 1 participant. ** Missing Data for two participants ↑ HADS Total / HADS Depression Patients n = 31, Overall (Patients + Healthy Controls) n = 48, HADS Anxiety Patients n = 33, Overall n = 50, Ø BDI-II Patients n = 29, Overall n= 45

### Exploration of resilience and psychological well-being in Friedreich ataxia

Patients with Friedreich ataxia showed lower scores on the RS-25 (135 ± 25) than healthy controls (151 ± 12). Cohen’s d was d = 0.73, representing a moderate effect size. A strong inverse correlation was observed between resilience scores and total HADS scores in patients with Friedreich ataxia, r(29) = -0.806, p < 0.001. Similarly, resilience scores exhibited a significant and strong inverse correlation with the HADS-Anxiety subscore, r(31) = -0.643, p < 0.001. Furthermore, the depression subscore demonstrated an even stronger significant inverse correlation with resilience scores than with the total HADS score, r(29) = -0.85, p < 0.001. The BDI-II total score also showed a significantly strong negative correlation with the RS-25 score in patients with Friedreich ataxia (r(27) = -0.788, p =  < 0.001). Univariate analysis revealed no demographic, clinical, lifestyle-related, or socioeconomic factors significantly associated with resilience after Bonferroni correction (Table 2) . However, some variables – such as wheelchair dependence, shorter GAA repeat length, psychiatric comorbidity, and partnership status – showed moderate effect sizes and nominal significance. These trends may reflect underpowered analyses rather than true absence of effect.

**Table 2:** Results of univariate analysis

	Pearsons correlation		Model summary (median)		Coefficients						N
Variable	Correlation	p	R2*	adj R2*	B	95% Confidence intervall		beta*	t	p-Value	
Demographics											
Age at Visit	0.24	0.06	0.06	0.03	0.51	–0.14	1.16	0.24	1.55	0.12	43
Sex (Male)	0.14	0.18	0.02	0.00	7.19	–8.16	22.54	0.14	0.92	0.36	43
Social											
No Employment	–0.03	0.43	0.01	–0.02	–1.40	–17.25	14.46	–0.03	–0.17	0.86	43
No Partner ɫ	–0.30	0.03	0.09	0.06	–15.27	–30.42	–0.12	–0.30	–1.98	0.05	43
ISCED Levels	0.25	0.06	0.06	0.04	6.09	–1.19	13.37	0.25	1.64	0.10	42
Clinical											
ADL Sum	–0.19	0.11	0.04	0.01	–0.68	–1.75	0.39	–0.19	1.25	0.212	43
Age at Symptom Onset	0.18	0.12	0.03	0.01	0.72	–0.48	1.91	0.18	1.18	0.239	42
No/Mild Disability	0.03	0.44	0.01	–0.24	3.02	–33.32	39.35	0.03	0.16	0.871	43
GAA Repeats Shorter Allele	–0.30	0.02	0.09	0.07	–0.03	–0.07	–0.01	–0.30	–2.04	0.041	43
SARA Score	–0.19	0.12	0.03	0.01	0.50	–1.34	0.33	–0.19	–1.18	0.238	41
Scoliosis	–0.01	0.48	0.00	–0.03	–0.46	–20.23	19.32	–0.01	–0.05	0.964	42
Wheelchair Bound	–0.34	0.01	0.11	0.09	–16.77	-31.25	–2.29	–0.33	–2.27	0.023	43
Psychiatric Illness**	–0.29	0.03	0.09	0.07	–18.30	-36.33	–0.30	–0.29	–1.99	0.047	43

*imputational average, bonferroni adjusted p-value: 0.05/13 = 0.004. ** assessed through medical history ɫ includes relationship status single, widowed, divorced, separated; partner group includes relationship status married, in a relationship. B = unstandardized coefficients, Beta = standardized coefficients

### Longitudinal assessment

**Fig. 1:**
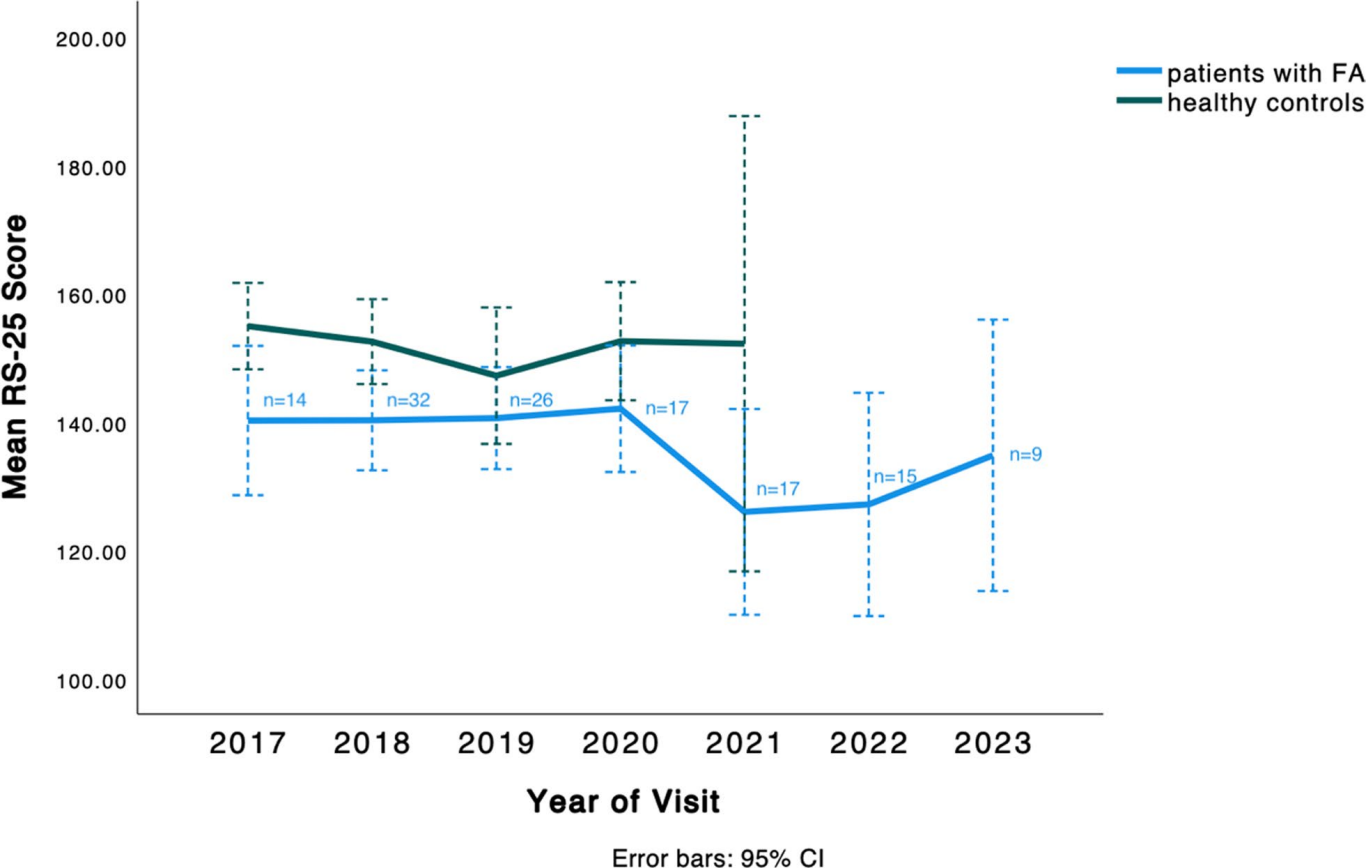
Longitudinal development of yearly mean RS-25 scores, Data represent a combination of cross-sectional and within subject observations. See Table S1 for quantification of scores

We assessed the longitudinal development of yearly mean RS-25 scores of patients with Friedreich ataxia and humans without Friedreich ataxia over six- and four-year periods (2017—2023 and 2017—2021), respectively (Fig. 1). The longitudinal trends depicted in Fig. 1 are based on a combination of cross-sectional and limited within-subject data rather than a fixed cohort followed longitudinally. Owing to the COVID-19 pandemic, the assessment of healthy controls paused between Spring 2020 and Summer 2021, whilst the assessment of patients continued.

Resilience Scores for patients with Friedreich ataxia were relatively stable from 2017 to 2020 but decreased during the COVID-19 pandemic, with mean scores falling substantially from 142 in 2020 to 125 in 2021 and remaining low at 126 points in 2022 (Fig. 1 & Figure S1). In 2023, the scores increased to 134 points but did not match the pre-pandemic scores.

18 participants had data on at least two visits. The mean resilience score in this subset before COVID-19 was 143.1 (SD = 22.2) and the mean score after COVID-19 was 131.2 (SD = 30.18), yielding a mean difference of 11.9 (95% confidence intervals -1.1, 25): p = 0.07.

**Fig. 2:**
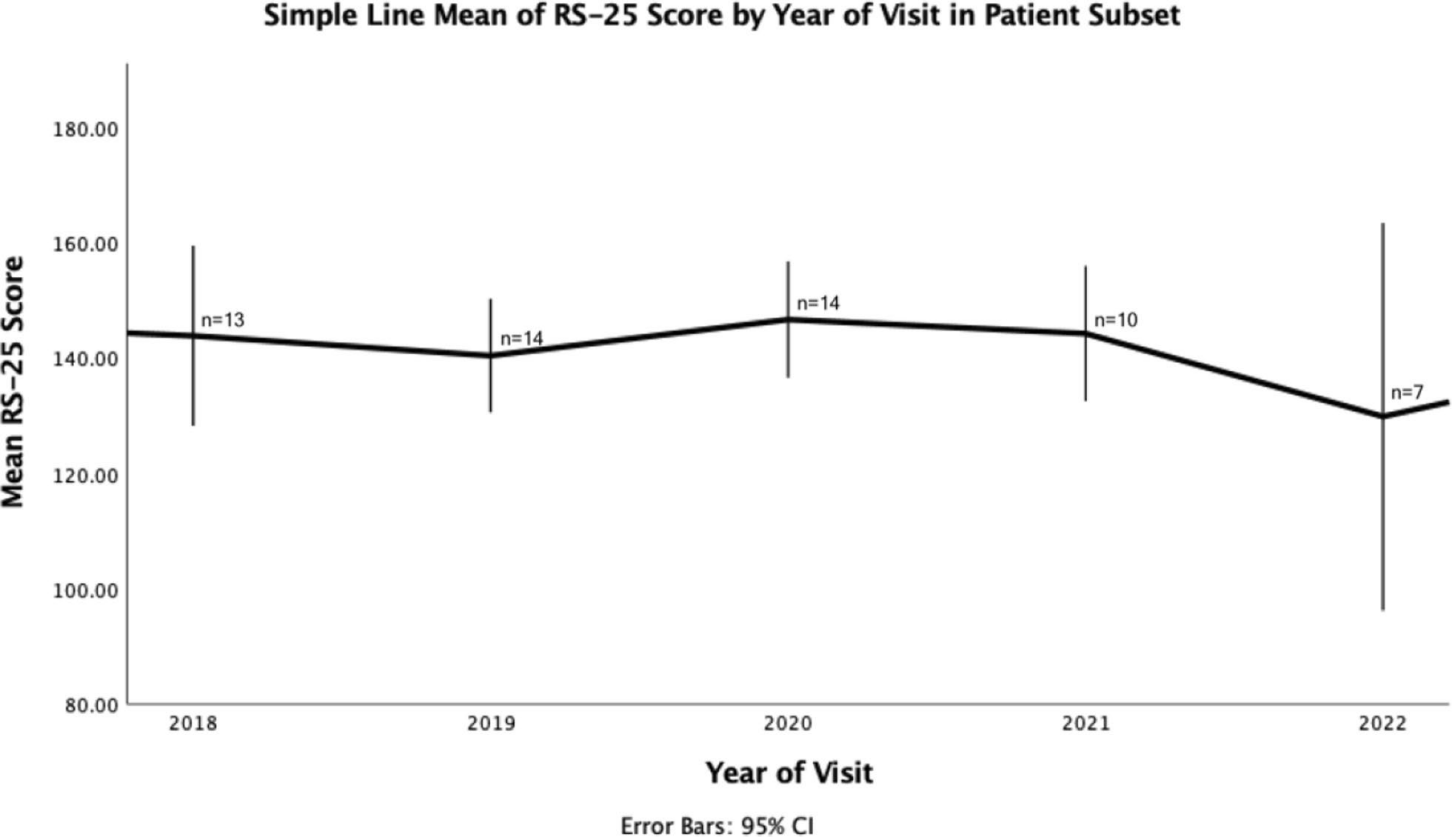
Longitudinal development of yearly mean RS-25 Scores in a subset of patients with Friedreich ataxia, Data represents within subject observation of a subset of patients with Friedreich ataxia with continuous data from 2019 to 2020, with at least 4 visits. Black line represents mean of RS-25 scores in that subset. See Table S2 for quantification of scores

**Fig. 3:**
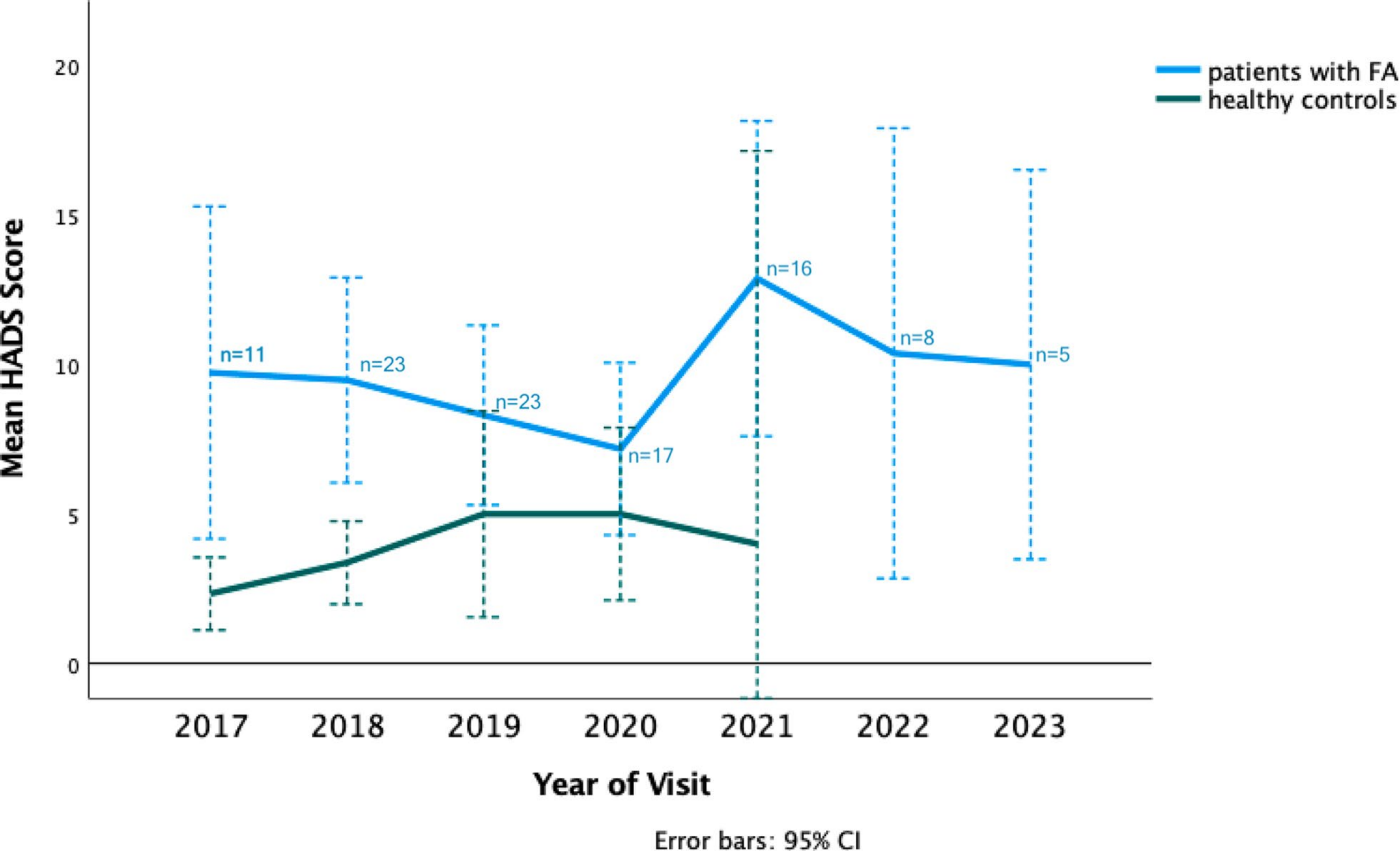
Longitudinal development of yearly mean HADS scores, Data represents a combination of cross-sectional and limited within subject observations. See Table S3 for quantification of scores

We also performed a graphical within-subject analysis of a subset of patients who have data available for both 2019 and 2020, with at least 4 visits (Fig. 2 & Figure S2). Within this small subset of 14 people with Friedreich ataxia, resilience scores remained largely stable. Additionally, we analysed the development of mean HADS scores on both cohorts over six and four years (Fig. 3). Mean HADS scores for patients with Friedreich ataxia decreased from 2017 to 2020, going from 9.73 to 7.8 respectively, but increased by about five points from 2020 to 2021. Mean HADS scores decreased again after that, going from 12.9 points in 2021 to 10 points in 2023 (Fig. 3 & Figure S3). We also assessed the development of resilience scores over the course of the visits.

## Discussion

In this longitudinal study, we investigated resilience in patients with Friedreich ataxia and healthy controls. Our research revealed substantially lower resilience scores in patients with Friedreich ataxia compared to healthy controls, as measured by the RS-25. Resilience scores also showed a strong inverse correlation with depression and anxiety assessment scores, indicating that higher resilience is associated with lower levels of both depression and anxiety. Resilience levels in individuals with Friedreich ataxia appeared to decline during the COVID-19 pandemic, which may reflect the impact of prolonged stress, albeit a within-subject subgroup analysis did not yield a statistically significant result, possibly due to low sample size. Concurrently, increases in mean HADS scores during the same period indicated a rise in depressive and anxious symptoms within the patient population. Finally, our analysis did not identify factors significantly associated with resilience among demographic, clinical, and social variables, although some weak to moderate, nonsignificant relationships such as wheelchair dependence, shorter GAA repeat length, psychiatric comorbidity, and partnership status were noted.

Compared with healthy controls, patients with Friedreich ataxia presented significantly lower resilience scores. This finding aligns with prior research, which has shown reduced psychological resilience in individuals with chronic conditions, notably other neurodegenerative diseases [18]. The unique challenges faced by patients with Friedreich ataxia, including reduced mobility, social isolation, and decreased quality of life may explain these findings [10, 31]. Unlike people without Friedreich ataxia, these patients often contend with persistent stressors that might hinder adaptive coping [32]. From a clinical perspective, these insights highlight the importance of incorporating resilience-building strategies, such as cognitive‒behavioural therapy, mindfulness practices, and social support programs, into patient care to enhance psychological well-being [33, 34]. In our study, the controls achieved a mean resilience score of 151 (± 12), which is consistent with values reported in a larger sample by Hector et al. (148 ± 15.6) [35]. In a review of the use of the RS-25 in a range of populations, Wagnild gives a normative range of RS-25 scores of 140—148 [36]. This suggests that the observed resilience levels are representative of comparable healthy cohorts in the literature. In contrast, a study investigating the stability of resilience during the COVID-19 pandemic reported considerably lower pre-pandemic (T0 = December 14, 2019 to March 10, 2020) baseline resilience scores in the German general population (134 ± 20) [37]. Substantially lower baseline resilience scores may reflect differences in recruitment strategy, demographic composition, and the timing and context of baseline assessments between the studies. The study’s cohort was predominantly female (82%) and highly educated employees with recruitment conducted through online enrolment and snowball sampling [37]. This approach may have introduced a self-selection bias. Resilience in the general German population remained relatively stable throughout the period assessed (December 14, 2019 to May 8, 2020) [37]. A comparable pattern could potentially be observed in our Friedreich ataxia cohort: resilience scores remained relatively stable between 2019 (pre-pandemic) and 2020 (early pandemic).

Conversely, resilience scores in the full cohort declined during the COVID pandemic, indicating a potential delayed or cumulative impact of prolonged pandemic-related stressors. This pattern should be interpreted cautiously, as it reflects cohort-level trends rather than individual-level changes*.* A pre- and post-COVID within-subject analysis in a small subset of patients with Friedreich ataxia suggests that resilience scores decreased during the pandemic albeit this decrease was not statistically significant, potentially due to low sample size.

Similar patterns have been documented in other populations, including healthy individuals, during this time [38]. However, such effects may be more pronounced in individuals with a chronic, progressive neurological condition, for whom ongoing disease burden, disruptions in healthcare access, and sustained psychosocial stressors may influence longer-term resilience trajectories. Our findings emphasize the necessity of targeted support during such disruptions [39]. For patients with Friedreich ataxia, the pandemic may have exacerbated existing challenges, including increased social isolation and disrupted access to care. Interventions such as virtual support networks, telemedicine services, and tailored mental health resources might have proven helpful in navigating these challenges. Building infrastructure for such measures now could mitigate the impact of future crises on mental health and resilience.

The strong inverse correlation between resilience and depression and anxiety observed in our study underscores resilience as a critical protective factor against symptoms of both disorders. The directionality of this relationship remains unclear, but our finding is consistent with previous research that identified resilience as a buffer for mental health challenges across diverse populations, including those with chronic illnesses [18, 40]. Strengthening resilience in patients with Friedreich ataxia could provide dual benefits: improving mental health outcomes and enhancing overall quality of life [19, 41]. Given the heightened vulnerability to depression in this population [9], resilience-focused interventions require more attention in clinical care. Research highlights resilience can be enhanced through a spectrum of interventions, ranging from informal practices such as meditation, social engagement, and educational activities to more structured approaches, including cognitive behavioural therapy [42]. Future research investigating the mechanisms by which resilience mitigates depression, particularly in hereditary neurological conditions, might be valuable.

Although we considered the possibility that demographic and clinical factors such as sex, age, ataxia severity, education level, and partnership status might shape resilience, our results did not support any clear associations. Factors such as GAA repeat expansion size, wheelchair use, and lack of a partner showed weak to moderate effects. We specifically expected ataxia severity, which is often linked to lower quality of life and loss of independence [10, 31], to correlate with resilience, potentially mediated by depression inversely. Our findings align with a meta-analysis showing only weak associations between age, sex and resilience in the general population [43]. Conflicting evidence exists regarding the relationship between depression and ataxia severity in patients with Friedreich ataxia with one study reporting a significant correlation [9] and another finding no linear relationship [44]. In other hereditary ataxias, such as spinocerebellar ataxias, depression is more clearly related to disease severity, with more severe ataxia linked to a higher likelihood of clinically relevant depression [45, 46].

The lack of significant associations in our study suggests that resilience in this population may be independent of demographic factors such as age and sex, as well as life circumstances such as relationship status, employment, or education level. Research in other neurological conditions highlights the importance of social support for mental well-being, with factors such as self-efficacy, optimism, and positive affect as key contributors to resilience [43]. Further research into the predictors of resilience may help identify target groups and inform the development of effective interventions.

### Strengths and limitations

The strengths of this study include the long follow-up duration of 6 years and the use of two scales, the HADS and the BDI-II, to evaluate depressive and anxious symptoms in patients with Friedreich ataxia. Despite these strengths, several important limitations exist. Owing to the rarity of Friedreich ataxia and the substantial proportion of the research period being in the global COVID-19 pandemic, resulting in cancellations of study visits in some cases, our sample size was small. Similar limitations exist regarding our control group; sample size was generally small and there were missing longitudinal data due to the COVID-19 pandemic. This limits our ability to generalize our findings to a broader population and increases the risk of type 2 errors. We therefore drew on previous literature on resilience in healthy controls for comparison. While our findings suggest an apparent decline in resilience during the COVID-19 pandemic, this observation is based on cohort-level trends. The subgroup with available within-person longitudinal data was small in size, limiting definitive conclusions about individual-level effects. Additionally, while our control group was matched with our cohort of people with Friedreich ataxia for age and sex it lacks matching in education, employment and relationship status. However, we did not find these factors to significantly impact resilience in our univariate analysis. Furthermore, owing to time constraints, not all patients attending our site participated in the resilience assessments, which may have introduced selection bias. The RS-11, a shorter version of the RS-25, could be considered to decrease patient and investigator burden. Furthermore, the BDI-II has items that assess somatic symptoms such as tiredness, which are common in patients with Friedreich ataxia but are not necessarily indicative of depression. To mitigate this, we primarily used the HADS scale, which does not assess somatic symptoms; it was not found to be superior to other scales in identifying emotional disorders in patients with a physical illness [47].

## Conclusion

Patients with Friedreich ataxia are less resilient than controls and thus may be more susceptible to mental health challenges. To the best of our knowledge, this is the first study to specifically examine resilience in people with Friedreich ataxia. While the lack of significant predictors limits the identification of specific at-risk groups, the observed relationship between resilience, depression and anxiety underscores a critical area for intervention. Programs aimed at enhancing resilience could improve both mental health and overall quality of life for these patients [33]. The COVID-19 pandemic was associated with an apparent decrease in resilience in this patient population, albeit not statistically significant in a subgroup with available within-person longitudinal data, highlighting the need for further investigation into resilience-focused strategies to support mental well-being during crises. Addressing factors such as social support, self-efficacy, and optimism can help mitigate the adverse effects of crises on vulnerable populations.

## Supplementary Information

Additional file 1.

Additional file 2.

Additional file 3.

## Data Availability

The datasets used and analysed during the current study are available from the corresponding author on reasonable request.

## Abbreviations

EFACTS: European Friedreich ataxia consortium for translational studies
RS-25: Wagnild and young resilience scale
HADS: Hospital anxiety and depression scale
BDI-II: Beck’s depression inventory II
IQR: Interquartile range
COVID-19: Coronavirus disease 2019
